# Pregnant women’s needs regarding the Tdap vaccine: Insights from Reddit discussions

**DOI:** 10.1371/journal.pgph.0006924

**Published:** 2026-08-12

**Authors:** Soojung Jo, Tatiana R. Ringenberg, Jon W. Hurley

**Affiliations:** 1 School of Nursing, Purdue University, West Lafayette, Indiana, United States of America; 2 Computer and Information Technology, Purdue University, West Lafayette, Indiana, United States of America; 3 Department of Computer Science, Purdue University, West Lafayette, Indiana, United States of America; PLOS: Public Library of Science, UNITED STATES OF AMERICA

## Abstract

Tdap vaccination during pregnancy is essential to protect the fetus, yet the vaccination rate in the United States remains suboptimal. Little is known about pregnant women’s perspectives or the factors that influence their Tdap vaccination decisions. Given that many pregnant women turn to social media and online sources for health information, this study aims to analyze social media posts from Reddit to better understand the needs of pregnant women regarding the maternal Tdap vaccine. This qualitative study analyzed the posts from Reddit collected from October 2020–2023. After screening, 86 posts were included for analysis. Thematic analysis was conducted. Four key themes emerged: information seeking, vaccine hesitancy, regulation and public policy, and concerns related to contact with newborns. Pregnant women actively sought information to clarify concerns and better understand healthcare providers’ advice. Some reported a lack of vaccine recommendations from providers, and inconsistent recommendations for the vaccination timing by the providers. Vaccine hesitancy and refusal were also expressed. In addition, confusion arose around policies recommending Tdap for family members and close contacts, leading to interpersonal conflict and resistance. Many posts described pushback from family members. Pregnant women face multifaceted needs and challenges related to vaccine information, policies, and emotional support. Healthcare providers should initiate the vaccine recommendation with clear information, considering the patients’ literacy and address concerns. Additionally, public health agencies and professional organizations should prioritize promoting the maternal Tdap vaccine by supporting access, including cost coverage.

## Introduction

The tetanus toxoid, reduced diphtheria toxoid, and acellular pertussis vaccine (Tdap) vaccination is crucial for pregnant women to protect both mothers and the fetus. Since infants under 2 months are too young to be vaccinated themselves [1], it is recommended that pregnant women receive the Tdap vaccine during pregnancy to transfer protective antibodies to the fetus [2]. Research shows that maternal Tdap vaccination significantly reduces the incidence of pertussis in infants younger than 2 months compared with infants aged 6 months to 12 months [3]. The United States (U.S.) Centers for Disease Control and Prevention (CDC) highly recommends that pregnant women receive the Tdap vaccine in their third trimester, and increasing vaccination rates among this population is part of the Healthy People 2030 goals [4]. However, despite its importance, Tdap vaccination rates among pregnant women in the U.S. remain suboptimal, with only 55.4% estimated to have received the vaccine in 2023 [5]. This contrasts with much higher vaccination rates for the childhood DTaP (diphtheria and tetanus toxoids and acellular pertussis vaccine) in the U.S., which is essentially the same as Tdap but formulated for a younger age group. The DTaP vaccination rate is 93.8% for infants by 24 months of age, among those born between 2019 and 2022 [6].

There is limited research on how to increase pregnant Tdap vaccine uptake; however, previous studies have identified several factors associated with maternal Tdap vaccine uptake, including receiving the provider recommendation and educational materials [7]. These findings suggest that providing accurate and accessible information may help address concerns and improve understanding, ultimately leading to increased vaccination rates. Although evidence specific to Tdap vaccine attitudes is scarce, a narrative review study on general vaccine acceptance among pregnant women showed that uptake is influenced by worries about potential side effects, lack of confidence in the vaccine, and a low perceived risk of infection, all of which are often rooted in insufficient knowledge [8]. Despite this, little is known about the specific information that pregnant women actively seek or what they want to know regarding the Tdap vaccine. Understanding these needs is critical for designing effective educational interventions and improving vaccine uptake.

Social media has become a frequently used platform for health information. An analysis of the national survey in the U.S. discovered that pregnant women increasingly rely on online sources for health-related information compared to a decade ago [9]. Pregnant women are an active group when it comes to searching for information about pregnancy and childbirth. A review study reported that pregnant women commonly use websites, social media, and smartphone applications for pregnancy-related information [10]. Specifically, research in the Netherlands found that pregnant women who feel socially supported are more likely to use digital sources such as websites and mobile applications [11,12]. In the context of vaccines, online platforms play a significant role. One study found that 61% of participants searched for information about the Tdap vaccine during pregnancy, with 76% relying on the internet or social media, which is more than those who consulted healthcare providers (63%) [13]. These findings highlight the importance of online platforms in addressing vaccine-related information needs for pregnant women.

Reddit is a widely used social media platform in English speaking countries that allows users to engage in anonymous discussions, offering an open space for people to freely express their thoughts without a word limit. This makes Reddit particularly suitable for in-depth discussions, and many studies have leveraged it to explore deeper insights from user conversations in health-related topics [14]. Given that health information is often considered private, people feel more comfortable sharing sensitive details anonymously on social media than with their healthcare providers [15]. In contrast to other social media platforms such as Facebook or Instagram, where real-name identities may limit the sharing of sensitive health information, or Twitter/X and TikTok, where short-form content constrains in‑depth discussion, Reddit’s anonymous and long-form, threaded structure provides a uniquely suitable environment for rich qualitative data collection [16,17]. Additionally, Reddit's main user base falls within the 18–29 age group, followed by 30–49, [18] which closely aligns with the typical age range of pregnant women. Therefore, Reddit provides a valuable opportunity to explore the specific information needs and concerns of pregnant women regarding the Tdap vaccine. Consequently, this study aims to analyze Reddit posts to better understand these needs. We seek to answer the question: What are the needs of pregnant women regarding the maternal Tdap vaccine?

## Materials and methods

### Study design

This is a qualitative study utilizing secondary data from the social media platform, Reddit. The study was conducted after receiving Institutional Review Board (IRB) exemption approval (IRB no. IRB-2023–697).

### Data collection

The data was collected using the Reddit API, adhering to the guidelines and restrictions of the Reddit API as of October 2023. We searched Reddit for posts written in English that contained the combination of two keywords: (*Tdap* (or *pertussis* or *whopping cough*) and *pregnant*) within the post title or body of the post. In October of 2023, we used the Reddit API to search through three years of Reddit data from October 11, 2020, to October 11 2023. We chose to search across all subreddits rather than restricting data collection to a small number of pregnancy or vaccine-focused communities. This decision was consistent with our aim to capture a wide range of perspectives from pregnant individuals discussing the maternal Tdap vaccine on Reddit, regardless of which specific community they chose to post in. Because our inclusion criteria were already highly specific (described in the next paragraph) and the Tdap vaccine for pregnant women is relatively new, expanding the search to the entire platform increased the likelihood of identifying relevant posts while still yielding a coherent, focused dataset. During our search, we collected no personally identifiable information about the individual who posted, instead focusing on the title and body of the posts. A total of 173 posts were retrieved.

### Data analysis

The initial screening of posts was done by the first author. The inclusion criteria for posts included: 1) the post was from the perspective of the pregnant women, 2) the post was made before their delivery, and 3) the maternal Tdap vaccine was the primary concern of the post. Although the inclusion criteria were straightforward, some of the posts from our search did not meet the full inclusion criteria. These posts were excluded during screening, but this process may introduce potential selection bias based on the interpretation of relevance. To reduce this potential bias, if the posts were ambiguous or unclear, all three researchers discussed and reviewed the post to make a final decision. As a result, 86 postings were screened and included in the data analysis.

We analyzed the data using reflexive thematic analysis [19]. Our approach involved familiarization of the coders with the data, generating initial codes, developing and reviewing candidate themes, identifying subthemes, and refining and naming the final themes and subthemes.

All three researchers conducted each step. First, familiarization with the data involved all authors reading through the entire dataset. Next, initial codes were then generated inductively by the authors based on the content of the posts. Coding was conducted independently using the open-source online tool Taguette [20]. During the coding process, the authors met frequently to discuss code insights, compare interpretations, and refine their coding approach, often drawing on the first author’s subject-matter expertise in vaccines and maternal health. After initial coding was complete, the authors merged similar codes and organized codes into broader candidate themes that captured insights relevant to the research. The authors discussed candidate themes in detail and compared all candidate themes against their associated coded excerpts to ensure themes were coherent and representative of the data. Within each candidate theme, we further examined the code excerpts to identify specific and consistent patterns within themes. When these patterns were distinguishable from other data and patterns within the theme, we identified them as subthemes to provide a more nuanced representation of the patterns present within an overarching theme. Finally, we refined and named our themes and subthemes to clearly convey the patterns relevant to the research.

Representative excerpts in the results were selected to illustrate the key themes identified in the analysis. The first author initially identified excerpts that were clear, detailed, and representative of the broader patterns observed in the dataset. The second author then reviewed these excerpts to ensure that the selected quotes reflected a range of perspectives and did not over‑represent any single type of post. Only quotes that directly supported the analytic claims and contributed to the clarity and credibility of the findings were included in the manuscript.

### Researcher positionality

The first author is an experienced health science researcher with both clinical and research backgrounds in infectious diseases, including conducting qualitative studies. The second author has substantial expertise in identifying language used in online manipulation and qualitative research using online data sources. The perspectives informed by these professional backgrounds may shape how the data are interpreted; therefore, the research team engaged in ongoing reflexive discussions throughout the analytic process to critically reflect upon, and mitigate, potential interpretive bias.

## Results

We identified four main themes, including information seeking, vaccine hesitancy, regulation and public policy, and concerns related to contact with newborns. Within the themes, we also identified ten sub-themes. Table 1 provides an overview of the themes, sub-themes, and the prevalence of excerpts supporting each sub-theme within a theme.

**Table 1:** Thematic findings and frequency of excerpts.

Theme	Sub-theme	Number of Excerpts
Information Seeking	Information Confirmation	4
	Information Needs	5
	Medical Advice	10
	Question About the Fetus	8
	Timeline for Vaccination	13
Vaccine Hesitancy	Safety	24
	Vaccine Refusal	7
Regulation and Public Policy		6
Concerns Related to Contact with Newborns	Pushback	44
	Rules and Boundaries	19

### Information seeking

Pregnant women actively sought information to address their concerns and clarify advice from their healthcare providers (HCPs). Their information-seeking behavior highlighted a desire for clarity, reassurance, and better communication about vaccination during pregnancy. Further, much of information seeking was driven by a desire to understand the timeline associated with vaccination, which served as the most prevalent subtheme of information seeking.

#### Timeline for vaccination.

Posts regarding the timeline of vaccination had the highest number of excerpts within the theme, ‘Information Seeking’. Questions regarding when the vaccine should be received often focused on the current efficacy of an older vaccine, as in the following example:

*“I’m wondering does a 6 year old tdap shot provide any antibodies during pregnancy”*

Other questions focused on the need for an additional vaccine following a previous pregnancy or as part of childhood immunizations. Posters also wanted to know if repeat vaccinations were safe. Concerns around safety within information seeking were also coupled with the theme of C*oncerns Related to Contact with Newborns* and whether the pregnant individual’s family members and friends should get vaccinated after the birth of their first child. Questions regarding the appropriate time for vaccination often stemmed from competing information from various sources and concerns regarding the medical advice received from doctors, as is demonstrated in the following quote, in which a poster is discussing competing information between two providers:

*“My previous provider led me to believe that, since a booster is recommended every 10 years … I have a new provider, and she’s saying all my family and friends will need to be boosted again, even though they already have been in the past 2 years.”*

The above quote demonstrates the tension caused by such varying advice. Such tension and skepticism were present across the timeline subtheme.

#### Information confirmation.

As shown with questions regarding the timeline of vaccination, some pregnant women expressed concerns with medical advice originating from multiple sources, including their HCPs, the Internet, and friends. Beyond concerns about timing, common post topics in which posters sought confirmation of information about the Tdap vaccine on Reddit included whether they should receive the Tdap vaccine, the differences between Tdap and DTaP, and a lack of definitive input from their own doctors.

In the following quotes, both posters describe the lack of definitive input received from their own providers:

*“What do you think about Tdap shot? … I asked my OB about my situation, he says it is up to me,…”*

*“ My provider hasn’t offered the tdap vaccine to me yet and I’m 36 weeks pregnant. Is it too late to get it now? … I switched providers in the late 2nd trimester… was reading that tdap is usually offered by now and with my baby being due soon… Is it too late to get it now?”*

The first poster indicates a vague response, placing agency on the decision of receiving the vaccine on the pregnant individual. In the second post, the individual describes a similar lack of guidance, which was inconsistent with the individual’s expectations.

#### Information needs.

The information needs subtheme focuses on posters seeking external validation, whether through hypotheticals, requests for online resources, or discussions around such resources.

The following excerpt comes from a poster who was told a Tdap vaccine was not necessary following a previous vaccine before pregnancy. As a result, the poster actively seeks to verify this information online:

*“my doctor …concluded that I didn’t need the TDAP during my pregnancy since I got it right before. Now I’m nervous that my baby won’t be protected… I’ve been looking online and can’t find any resources or guidance about this specific situation.”*

Lack of research or general information was seen across multiple posters, as in the following example, in which a poster seeks research on vaccine recommendations for families based on prior reading:

*“… I’m bummed there’s not great data on the effects of it. Does anyone have research that highlights the benefits of cocooning?(making family members receive the vaccine)... From what I’ve read, that’s the most effective way to protect the baby.”*

Through these posts, posters sought reassurance from formal and informal data sources as well as others in the Reddit community with similar experiences.

#### Medical advice.

In addition to posters seeking reassurance through information resources, posters also sought specific medical advice posts based on their own unique situations, often describing interactions with providers or descriptions of concerns in detail. The advice sought in these posts was beyond what was outlined in standard guidelines, and in several cases was driven by a need for information confirmation due to a lack of guidance or competing guidance between providers. Even when their HCPs offered advice tailored to their situations, many women felt frustrated, confused, or struggled to understand the explanations, thus turning to this online community for support. There were also posts indicating that a few women did not receive any recommendation from their HCPs and wanted to confirm whether the vaccine was necessary.

*“Got a tDap vaccine twice during pregnancy once during the first trimester before I knew I was pregnant. Then the doctor recommended it a second time at 28 weeks. I looked up the guidelines and it says the tDap should only be administered once during pregnancy. Anyone have any information about what this can do to baby?”*

*“… OB told me to ask a pediatrician (which I don’t have since my son isn’t born yet). I called a pediatrician’s office and they told me to ask my OB. Anyone know what to do in this particular situation?”*

#### Question about the fetus.

These excerpts reflect curiosity and concern regarding how the vaccine impacts their fetus, the duration of the protection it provides, and how immunity is transferred to their fetus. They also had concerns about potential allergic reactions to the vaccine:

*“I’m pretty pro vaccination, but I’ve never heard of vaccinating a heavily pregnant woman and also having that same dose transfer into a 29 week baby still in the womb. It sounds weird and dangerous to me.”*

*“If the mother knows she is not allergic to the TDaP, how does she know her fetus is not allergic to the TDaP… they have DNA from the father too.”*

### Vaccine hesitancy

Using the anonymity of the online platform, pregnant women expressed concerns about vaccine safety and discussed refusal of the vaccine.

#### Safety.

Safety was the second most quoted subtheme within the dataset. Pregnant women shared subjective concerns about potential side effects of the vaccine, including pain, palpitations, chest tightness, abnormal blood glucose levels, uterine contractions, swollen lymph nodes, and tenderness or swelling in the arm. Many also asked if others had experienced similar side effects or inquired about interactions with other vaccines, such as the Influenza or COVID-19 vaccines.

*“I just got the TDAP shot at my routine 28-week appointment on Friday… Anyway, I woke up yesterday to nausea, vomiting, diarrhea, and chills on and off all day. The major symptoms probably lasted about 12 hours… My question to you all is… has anyone else reacted this way to the TDAP while pregnant?*

*“I’ll be 28 weeks this Friday and I scheduled an appointment at my pharmacy to get the TDAP, flu, and COVID booster. Aside from the possibility of feeling sick, are there any reasons to not go for all three at once?”*

#### Vaccine refusal.

Some posters also wanted to understand whether they should avoid receiving the Tdap vaccine, questioning its necessity due to the perception that pertussis is eradicated. Many turned to this online community to seek advice that aligned with their existing preference to avoid vaccination, serving as a form of confirmation bias:

*“I’m 34 weeks pregnant. My doctor keeps urging me every appointment to get the Tdap. I deny it every time.”*

*“Has anyone refused the TDAP shot while pregnant. I’ve had some people tell me to be leery of it but just told me to do some research but I haven’t come across anything? So I’m confused.”*

*“Is it ok not to take whooping cough vacc during pregnancy?”*

These posts reflect the polarity of opinions regarding the Tdap vaccine in these online communities.

### Regulation and public policy

Some postings focused on the relatively recent recommendation for the Tdap vaccine during pregnancy, with multiple pregnant women questioning its necessity. The U.S. CDC advises that family members and caregivers stay up to date on vaccinations to form a protective circle of immunity around the newborn. This guidance led to frustration and confusion because the routine Tdap vaccine for adults is recommended once every 10 years. Pregnant women noted that insurance often does not cover the vaccine for family members unless they have not received the Tdap vaccine within the past 10 years.

*“I think I saw the CDC recommended it for pregnant women in 2012, but was it the same time frame for visitors? Just wondering since it seems fairly recent.”*

*“Now my husband is struggling to get his mom to get it (the vaccine) as well. Except in her case, she got it about 1 year ago for another infant in the family. She cited the cdc website which simply says adults around an infant who already had the shot don’t need to get it again before the 10 year window… My question is where to draw the line on this one.”*

As demonstrated in the quotes above, confusion regarding recommendations of the CDC could cause both confusion and concerns related to, and by, family members.

### Concerns related to contact with newborn

The majority of postings discussed conflicts with close family members who would have contact with their newborns. Many pregnant women faced resistance from their families regarding the Tdap vaccine, which often resulted in significant stress for the poster and hesitation regarding enforcement of vaccination.

#### Pushback.

Pushback of family and friends against vaccination was the most prevalent subtheme we found in the dataset. Many posters shared experiences of dealing with family members who were opposed to vaccinations, particularly anti-vaxxers. Some also encountered resistance to the Tdap vaccine within their own households, facing a lack of support from their husbands. By sharing these challenges on platforms like Reddit, they sought advice on how to navigate such conflicts.

*“Husband and I have a disagreement … our newborn seeing an unvaccinated person… But I feel like it isn’t as high compared to having our newborn see an unvaccinated relative who does not want to get the flu, TDAP, and Covid vaccines… I view a newborn getting whooping cough or a fever more severe than me eating deli meat. What are your thoughts?”*

*“I don’t feel comfortable with having anyone around my baby who doesn’t have the tdap vaccine. Whooping cough is no joke, and my obgyn recommends everyone who is going to be around my daughter should get it. My family has agreed, but my partners family is really anti vaxx.”*

#### Rules and boundaries.

Several posters attempted to put rules and boundaries in place for family and friends with respect to getting the vaccination and interacting with the newborn. These posters turned to online for advice on how to effectively communicate and enforce these rules.

*“I wanted to request that everyone be up to date on TDAP/Covid/flu shots prior to baby being here, as we are expecting both sets of parents and siblings to meet baby after birth… but my mom was giving me some pushback saying she doesn’t think I can request people getting vaccines… Has anyone felt like this? Is this normal or is this worth talking to my doctor about?”*

The above post demonstrates the difficulties pregnant women had when communicating boundaries and rules to family members who resisted vaccination, often with family members stating that vaccination was not appropriate to force on others.

In the following excerpt, a poster describes similar tensions which occurred with a family member following their initial agreement to comply with the wishes of the poster and the poster’s husband:

*“… But now MIL has changed her tune and is refusing (FIL is getting his). She said she’ll wear a mask if that makes me more comfortable but she will not be getting a shot. My husband and I are so frustrated… He’s going to talk to her tomorrow and lay out all of our rules and boundaries (we’re in total agreement over everything) for her to think over before making a definitive decision about the vaccine… Any rules we aren’t thinking of in terms of keeping baby safe?”*

As shown above, posters were not only faced with initial pushback but also family members attempting to negotiate the boundaries expressed by the poster, which led to continued stress and tension.

## Discussion

This study explored the needs of pregnant women regarding the Tdap vaccine by analyzing social media postings from Reddit. The findings revealed diverse and multifaceted needs, including information seeking, vaccine-related concerns, challenges related to policy and regulation, and emotional support related to their families. The results suggest that HCPs and government agencies should prioritize addressing these concerns to better support pregnant women in making informed decisions about the Tdap vaccine as well as their families.

One key area identified in this study is that pregnant women often felt confused by the information provided to them, or in some cases, by the lack of information or recommendations from their HCPs. Although their HCPs offered tailored recommendations based on individual circumstances, the lack of clear and consistent guidelines contributed to their confusion. The pregnant women in our dataset considered the guidelines, including the sources from the U.S. CDC, to not be clear enough, leading them to choose to go online to the Reddit community to seek assistance. Additionally, some pregnant women noted that their HCPs did not mention or recommend the Tdap vaccine at all, which further undermined confidence and contributed to confusion. The findings also revealed a lack of specific guidance for families, particularly regarding whether family members needed additional doses of the vaccine based on their prior vaccination history, further contributing to confusion. Even when pregnant women received accurate information, many struggled to fully understand the recommendation and its rationale, often turning to online platforms for additional clarity. Additionally, our findings emphasized the need for educational approaches or standardized guidelines tailored to the health literacy levels of pregnant women. Many struggled to fully understand the recommendation and its rationale, even though they were provided with correct information. Literacy-based education is essential for improving vaccine comprehension and uptake. Previous research from the Netherlands demonstrated positive outcomes from using online tailored decision aids to support pregnant women in understanding the Tdap vaccine [21]. Another study similarly indicates that providing clear explanations of the rationale behind vaccine guidelines increases knowledge and confidence [22]. Our results align with this evidence, indicating that simply providing accurate information is insufficient. Future efforts must focus on improving health literacy and providing decision-making tools that empower pregnant women to make informed choices.

Relatively few posts contained discussions of vaccine hesitancy or negative perceptions of the Tdap vaccine compared to other vaccines. Studies have consistently reported extensive negative information regarding vaccines like the Human Papillomavirus [23] or childhood vaccines [24]. This difference may stem from the perception that the Tdap vaccine is primarily intended to protect newborns, making the rationale for vaccination clearer for some individuals and acceptable to pregnant women. Nonetheless, safety concerns persisted among those who expressed hesitation. Based on these findings, future studies should further examine the nature of safety concerns among pregnant women, particularly what causes the hesitation. A systematic review has shown that actively involving patients in the decision‑making process can improve vaccine uptake [25]. Therefore, future research should focus on training HCPs in shared decision-making approaches and supporting pregnant women in actively participating in vaccination decisions.

Another important finding from this study is the multifaceted nature of care for pregnant women, particularly their efforts to protect their newborns. Many expressed a desire to set boundaries for close contacts, encouraging family members to stay up to date on the Tdap vaccine. However, persuading others to get vaccinated was far more challenging than making vaccination decisions for themselves, adding significant stress to pregnant women. This stress remains an underexplored area in both research and policy. Moreover, general awareness of the Tdap vaccine among adults is limited, with only 59.2% of adults reported to be up to date in 2022. [26] Public health campaigns should focus on raising awareness about the necessity of the Tdap vaccine, both for pregnant women and those in close contact with newborns, to enhance general understanding and alleviate the burden on pregnant women to educate and convince others.

The findings of this study highlight the need to address the multifaceted needs of pregnant women regarding the Tdap vaccine. Clear and consistent guidelines, along with health literacy-based ones, are essential to ensure that pregnant women can make informed decisions. Moreover, public health campaigns should emphasize the importance of the Tdap vaccine in protecting newborns, raising awareness among the general public to reduce vaccine hesitancy, and increase acceptance. Given the growing pushback against vaccination in public discourse, professional organizations such as the American College of Obstetricians and Gynecologists and the American Academy of Pediatrics may need to play an expanded role in providing clear, evidence‑based public health information to ensure that pregnant women and their families receive consistent and trusted guidance.

### Limitations

This study has several limitations. First, the analysis was based on secondary data from Reddit, which may not fully represent the views of all pregnant women. Reddit users may be more active information seekers than the general population, potentially limiting generalizability. Second, the posts analyzed were self-reported and could not be independently verified, which may affect the reliability of some claims. While Reddit’s community dynamics may help surface inconsistencies, the anonymity and informal nature of posts may still lead to incomplete or biased representations. Third, thematic analysis is interpretive by nature, and although multiple coders and consensus discussions were employed to enhance rigor, some degree of subjectivity is inherent in qualitative analysis. Next, because only English-language posts were included, the findings may not reflect exclusively U.S. perspectives. Finally, the study design did not allow for follow-up with participants to clarify or expand upon their statements, limiting the ability to probe deeper into individual experiences. These limitations should be considered when interpreting the findings, and future research incorporating diverse recruitment strategies, longitudinal designs, and direct participant engagement could help address these gaps.

### Clinical implications

The findings of this study highlight important implications for clinical practice, particularly in supporting HCPs in promoting maternal Tdap vaccination. Current research indicates that not all HCPs recommend the maternal Tdap vaccine, only 66.7% reported they provide timely recommendations [7]. Given that provider recommendation is consistently identified as one of the strongest predictors of vaccine uptake in general [27,28], it is essential that HCPs initiate Tdap discussions proactively and deliver comprehensive, accurate, and tailored information. Providing explanations that align with the pregnant woman’s health literacy level and addressing safety concerns, vaccine timing, policies, and the rationale for maternal immunization may help improve understanding and reduce hesitancy. Strengthening communication practices and ensuring that all pregnant women receive a consistent, well-informed recommendation may ultimately contribute to increasing Tdap vaccination rates.

## Conclusions

This study highlights several critical information needs among pregnant women regarding the Tdap vaccine, including confusion arising from inconsistent information, gaps in provider communication, and uncertainty about guidance for family members. Safety concerns and vaccine hesitancy also emerged as significant factors shaping decision-making. By leveraging real‑time, narrative data from Reddit, this study provides unique insight into the lived experiences and information gaps that may not surface in clinical settings or traditional surveys.

These findings highlight opportunities for improving both clinical practice and broader vaccine communication strategies. Clear and consistent provider recommendations, health‑literacy–appropriate education, and targeted messaging for family members and close contacts are essential components for increasing maternal Tdap uptake. Strengthening public health guidance and ensuring alignment across clinical and community-facing communication channels may further reduce confusion and support informed decision‑making.

Future research should explore interventions designed to enhance provider communication, address family‑level barriers, and evaluate the effectiveness of social-media–informed educational strategies. Addressing these multifaceted needs is critical for improving maternal vaccination and ultimately safeguarding newborns against pertussis.
